# Supplementary material for: Learning and Investigation of the Role of Angiotensin-Converting Enzyme in Radiotherapy for Nasopharyngeal Carcinoma
Source: Biomedicines. 2023 May 30;11(6):1581. doi: 10.3390/biomedicines11061581 (PMC10295367; doi:10.3390/biomedicines11061581)
Supplement: Supplementary file 1 [file biomedicines-11-01581-s001.zip › biomedicines-2364500-supplementary.pdf]

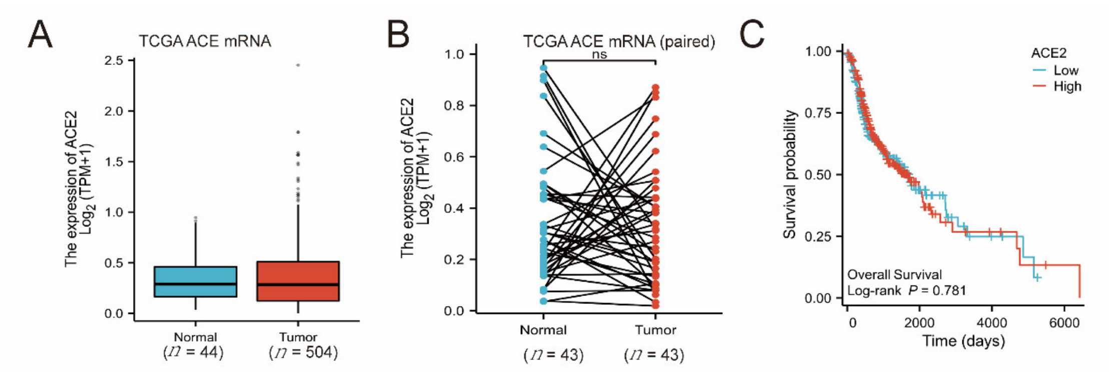

Figure S1. Expression of ACE2 in HNSCC dataset from the TCGA database. (A) Expression of ACE2 in HNSCC and normal tissue samples from the TCGA database. (B) 43 paired samples isolated from TCGA, HNSCC versus adjacent non-tumor tissues; expression level comparison of ACE2 in the 43 paired samples. (C) K–M analysis of overall survival between the high ACE2 expression group and low ACE2 expression group in HNSCC. Data were analyzed using Xiantao Academic Online Tools. ns, $p \geq 0.05$.
